# Supplementary material for: African Trypanosomiasis: Extracellular Vesicles Shed by Trypanosoma brucei brucei Manipulate Host Mononuclear Cells
Source: Biomedicines. 2021 Aug 20;9(8):1056. doi: 10.3390/biomedicines9081056 (PMC8394715; doi:10.3390/biomedicines9081056)
Supplement: Supplementary file 1 [file biomedicines-09-01056-s001.zip › biomedicines-1323143-supplementary.pdf]

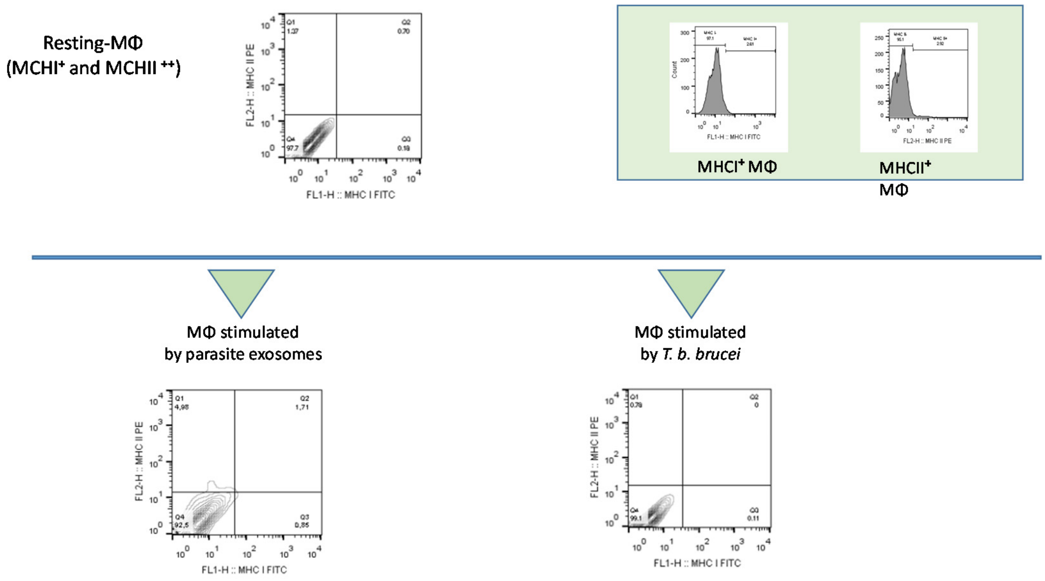

Supplementary Figure S1. Representative histograms and dot plots of MHCI and MHCII labelled macrophages analysed by flow cytometry. Unlabelled density plots and histograms are shown as well as quadrant gating plots presenting subset frequencies of MHCI and MHCII in resting-MΦ, parasite exposed MΦ, and TbEVs stimulate MΦ.

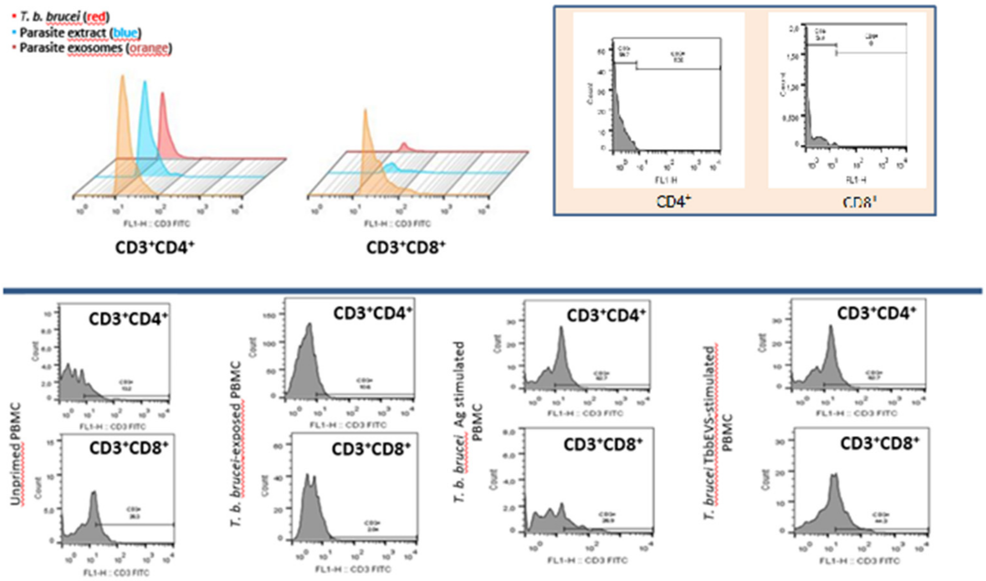

Supplementary Figure S2. Representative histograms of CD3 labelled CD4+ and CD8+ leukocytes were analysed by flow cytometry. Unlabelled CD4+ and CD8+ cell fractions are shown by histograms. The frequency of CD3+ in CD4+ and CD8+ cell fractions stimulated by Ag and TbEVs and exposed to *T. b. brucei* are represented by histograms.
